# Nitric Oxide Does Not Improve Liver Mitochondrial Function 48 Hours After Cecal Ligation and Perforation in Experimental Sepsis

**DOI:** 10.3390/antiox14070868

**Published:** 2025-07-16

**Authors:** Pierre Eyenga, Shey-Shing Sheu

**Affiliations:** Center for Translational Medicine, Department of Medicine, Sidney Kimmel Medical College, Thomas Jefferson University, Philadelphia, PA 19107, USA; shey-shing.sheu@jefferson.edu

**Keywords:** liver, CLP, mitochondria, sepsis, oxidative phosphorylation, nitric oxide, mitochondrial permeability transition pore

## Abstract

Nitric oxide (NO) has a dual effect on mitochondria. Incubating liver mitochondria with NO improves oxidative phosphorylation (OXPHOS) efficiency by decreasing state 4 respiration more than ATP synthesis and preventing mitochondrial permeability transition pore (mPTP) opening. We evaluated the effect of L-arginine (L-arg), an NO donor, on isolated liver mitochondrial respiration and mPTP in sepsis. Male mice were subjected to cecal ligation and perforation (CLP) with saline resuscitation or sham. After 8, 24, and 48 h, with and without L-arg, we measured isolated liver mitochondrial respiration and cytochrome c oxidase (COX) activity using polarographic methods and calcium retention capacity (CRC) to assess the mPTP and NO metabolites via the Griess reaction. Mitochondrial NO synthase (mtNOS) was identified by Western blot. CLP decreased state 3 respiration at 24 and 48 h, decreased COX activity at 8, 24, and 48 h, and increased state 4 respiration and decreased the respiratory control ratio (RCR) and CRC at 48 h. L-arg increased NO levels at 8 h, decreased state 4 respiration more than state 3 respiration (−39% versus −12%) at 48 h, decreased the CRC in the CLP groups at 24 and 48 h, but did not improve RCR. Our data suggests that L-arg does not restore liver mitochondrial OXPHOS efficiency or prevent mPTP opening in the late or recovery phases of sepsis.

## 1. Introduction

Sepsis and septic shock remain the leading causes of death in intensive care units despite numerous therapeutic trials [1]. After surviving the early shock phase [2], most patients die from sepsis-induced multiple organ failure (MOF) [3]. Because of its important role in metabolism and host defense mechanisms, liver dysfunction is thought to be central to the development of MOF during sepsis [4]. Although a pro-inflammatory response can lead to early liver failure [5], previous experimental studies have related initial liver dysfunction to microcirculation alterations following excess nitric oxide (NO) production [6] and/or early mitochondrial dysfunction [7], which appears to be an adaptive state [8]. Excess NO production from inducible nitric oxide synthase (iNOS) isoforms following cecal ligation and puncture (CLP)-induced sepsis is associated with impaired liver microcirculation [6,9] in addition to vasoplegia and cardiac dysfunction [10,11]. A mitochondrial isoform of nitric oxide synthase (mtNOS) was recently described [12,13]. Its activation could inhibit mitochondrial respiration and ATP production [14]. However, some authors have described a beneficial effect on mitochondrial function and oxidative stress in the brain cortex mitochondria of diabetic rats [15]. The local increase in NO following its activation could result in a delay in mitochondrial permeability transition pore (mPTP) opening [16] and an increase in mitochondrial oxidative phosphorylation (OXPHOS) efficiency [17]. This increase in OXPHOS efficiency was related to a kinetic constraint on cytochrome oxidase (COX), which decreased oxygen consumption more than ATP synthesis [17]. A large body of evidence indicates that COX is able to undergo slippage [17,18,19,20,21]. In the intermediate phase of experimental sepsis, this slippage results in a decrease in mitochondrial OXPHOS efficiency [19], contrary to hypothyroidism [20] or chronic ethanol ingestion [21] in rats. It is well-known that incubating liver mitochondria with a small amount of NO for a short period can lead to a decrease in COX slippage [17] and an increase in OXPHOS efficiency. Given that we expected an increase in mitochondrial efficiency in the late and recovery phases of sepsis [22,23], we hypothesize that the activation of mtNOS and the subsequent increase in mitochondrial NO production could decrease state 4 respiration more than state 3 respiration and thus increase the respiratory control ratio (RCR). This adjustment of mitochondrial OXPHOS efficiency could be the underlying cause of an improvement in mitochondrial function in the recovery phase of sepsis [22,23]. Here, we studied the functional parameters of mitochondria isolated from septic and control mice at 8, 24, and 48 h after CLP, particularly oxygen consumption, COX activity, the CRC, and the mPTP in the absence and presence of L arginine (L-arg), which is a well-known activator of mtNOS in vitro [14,15,16,24,25].

## 2. Materials and Methods

### 2.1. Surgical Procedure

#### 2.1.1. Animals

All animal experiments were conducted in accordance with experimental protocols approved by the Institutional Animal Care and Use Committee of Thomas Jefferson University. The animals were maintained under pathogen-free conditions (22 ± 0.5 °C, 55% relative humidity, and 12 h: 12 h light–dark cycle) at the Thomas Jefferson University facility.

#### 2.1.2. Cecal Ligation and Puncture (CLP)

CLP surgery was performed according to the protocol previously described, with some modifications [26]. This study used 8–12-week-old male C57BL/6 mice. In both the sham operation and CLP groups, anesthesia was induced using isoflurane inhalation at 2–4% and maintained during the surgical procedure. The abdomens were shaved, and a ~1 cm midline incision was made. The cecum was isolated and ligated below the ileocecal junction without causing bowel obstruction, punctured twice with a 20-gauge needle, and then gently squeezed to ensure the holes were patent. The cecum was placed back in the abdomen, and the incision was closed with sutures. All animals were resuscitated with a subcutaneous infusion of normal saline solution (10 mL·kg^−1^) immediately after surgery and then every 8 h. To alleviate discomfort, the animals received a subcutaneous injection of buprenorphine 0.05 mg/kg diluted in saline immediately after the surgery and then every 8 h. After the surgery, the mice had free access to water and food. Survival was monitored every day for 72 h. This protocol led to 75% mortality within 2 days [26,27,28], with most deaths occurring before 48 h. Additional matching animals were randomly assigned to undergo a sham operation as a control group (n = 18). The sham operation (control) involved laparotomy and cecal deliverance without ligation or puncture. Surviving CLP animals (n = 18) were matched with sham-operated animals at 8, 24, and 48 h post-surgery. In total, 68 mice were used in this protocol.

### 2.2. Liver Mitochondrial Isolation

Liver mitochondria were prepared according to standard differential centrifugation procedures, as previously described, in a medium containing 250 mM sucrose, 20 mM Tris/HCL, and 1 mM EGTA at a pH of 7.3 [7,19]. The protein concentration of the mitochondrial suspensions was determined using the biuret method, with bovine serum albumin as a standard.

### 2.3. Mitochondrial Respiration and Cytochrome C Oxidase Activity

Oxygen consumption was measured at room temperature using a Clark-type oxygen electrode from Hansatech (PP Systems, Boston, MA, USA) and was calibrated with an air-saturated respiratory buffer (120 mmol L^−1^ KCl, 5 mmol L^−1^ KH_2_PO_4_, 1 mmol L^−1^ EGTA, 2 mmol L^−1^ MgCl_2_, 0.3% fatty acid-free bovine serum albumin (*w*/*v*), and 3 mmol L^−1^ Hepes, pH 7.4). Liver mitochondria (1 mg mL^−1^) were incubated in the respiratory buffer, and 5 mmol L^−1^ of glutamate/2.5 mmol L^−1^ of malate was added to start the oxygen consumption. The active state of respiration (state 3) was initiated by adding 0.5 mmol L^−1^ of ADP. The basal non-phosphorylating respiration rate (state 4) was obtained by adding 3 µg mL^−1^ of oligomycin. The uncoupled respiration state was initiated by adding 2 µmol L^−1^ of carbonyl cyanide p-trifluoromethoxyphenylhydrazone (FCCP). Thereafter, antimycin (1.5 µmol L^−1^) was added to fully inhibit glutamate/malate-supported respiration. Then, ascorbate (2 mmol L^−1^) and TMPD (0.5 mmol L^−1^) were added, and the maximal respiration rate associated with isolated cytochrome c oxidase activity was recorded. RCR refers to the ratio of oxygen consumed after adding ADP compared to the amount consumed in the presence of oligomycin.

### 2.4. Mitochondrial Calcium Retention Capacity

Mitochondria (1 mg mL^−1^) were incubated at room temperature in 1 mL of a buffer (250 mmol L^−1^ sucrose, 10 mmol L^−1^ Mops, 1 mmol L^−1^ Pi-Tris, 0.15% FFA-BSA, and pH 7.4) containing glutamate (5 mmol L^−1^) and malate (2.5 mmol L^−1^). Changes in the extramitochondrial calcium concentration were monitored fluorometrically (Varian Peltier multicell holder, Agilent Technologies, Inc., Santa Clara, CA, USA) using 0.25 µM Calcium Green-5N (excitation: 506, emission: 530 nm), as described by Fontaine et al. [29]. The measurement started after 2 min of incubation, and Ca^2+^ pulses (15 µmol/mg protein) were successively added after 2 min of signal stabilization at 2 min intervals until mitochondrial permeability transition pore (mPTP) opening, as indicated by the release of Ca^2+^ into the medium. The results are expressed as the amount of Ca^2+^ added [30]. The increase in the CRC was quantified using 1 µmol L^−1^ cyclosporine A (CsA) as the competitive inhibitor of cyclophilin D (the referent inhibitor of mPTP).

### 2.5. Activation of Mitochondrial Nitric Oxide Synthase

The activation of mitochondrial nitric oxide synthase (mtNOS) was produced by incubating mitochondria with L-arg [14,24]. In line with our previous work [14], we used 1 mmol L^−1^ of L-arg to stimulate mtNOS. NG-nitro-L-arginine methyl ester (L-NAME) was used to inhibit mtNOS.

### 2.6. Measurements of Nitric Oxide Metabolites Level in Mitochondrial Pellet

After in vitro mitochondrial respiration was assessed, a respiratory buffer containing mitochondria was centrifuged at 13,000× *g* for 5 min. A 1 mL aliquot of a supernatant fraction was then used to determine the nitrite/nitrate levels (NOx-), as previously described [31]. Each sample was deproteinized with NaOH and incubated for 30 min at 30 °C in the presence of Cu-coated Cd to reduce NO3- to NO2-. A Griess reaction was then performed at room temperature for 5 min, and the optical density was measured at 540 nm. The results were expressed in micromoles of NOx- per mg of protein (µmol/mg protein). The protein concentration in supernatant fractions was determined using Bradford’s method.

### 2.7. Western Blot Analysis

To ensure that the inhibition of mitochondrial respiration followed mtNOS activation, we incubated 100 µG of mitochondria pellets with anti-iNOS using Western blot standard techniques, as described previously [6,7,19]. Because thymus and liver mitochondrial nitric oxide synthase (mtNOS) were found to react with anti-NOS 2 [13,32], we used the primary antibodies of anti-mouse polyclonal anti-iNOS to identify mtNOS. The primary antibodies employed were a mouse anti-mouse polyclonal anti-iNOS (1:1000 dilution; Invitrogen, Cergy Pontoise, France) and horseradish peroxidase-linked goat anti-rabbit secondary antibodies (1:10,000 dilution, containing secondary antibody goat anti-rabbit; Sigma Aldrich, Saint-Quentin Fallavier, France); these were used for mt NOS detection. Duck muscle mitochondria were used as a negative control.

### 2.8. Statistical Analysis

Stat view 5.01 for Windows (Informer Technologies Inc., Los Angeles, CA, USA) was used for statistical analysis. The Shapiro–Wilk test showed a skewed (i.e., not normal) distribution of variables. Thus, the variables were presented as the median [interquartile range (IQR)]. Differences in variable mean values between the groups were tested by a Mann–Whitney test. GraphPad Prism 10 (GraphPad Software Inc., San Diego, CA, USA) was used to draw the figures.

## 3. Results

### 3.1. CLP Decreases Mitochondrial Active Respiration and Cytochrome C Oxidase Activity

Phosphorylating mitochondrial respiration (state 3 respiration rate) was initiated by adding ADP in the presence of glutamate and malate as substrates. In the CLP groups, the median [interquartile range (IQR)] of the oxygen consumption of liver mitochondria was significantly reduced by 36% at 24 h (4.23 [1.33–4.70]) nmol O_2_/min/mg of protein (*p* = 0.0088) and 60% at 48 h (2.66, [2.43–3.70]) nmol O_2_/min/mg of protein (*p* = 0.0090) compared to the matching sham-operated control at 24 h (6.66 [5.13–10.02]) and 48 h (6.24 [6.08–9.62]), respectively. However, the difference was not significant in early sepsis (8 h) but showed time dependence throughout the course of sepsis when comparing the median [interquartile range (IQR)] of mitochondrial oxygen consumption at 48 h (2.66, [2.43–3.70]) nmol O_2_/min/mg of protein and 8 h (5.40, [3.26–6.55]) nmol O_2_/min/mg of protein (*p* = 0.0143) (Table 1). In the CLP groups, the median ([interquartile range (IQR)] of basal non-phosphorylating respiration measured in the presence of oligomycin (state 4 respiration rate) was significantly increased (+69%) only 48 h after the onset of CLP (1.41, [1.12–2.38]) nmol O_2_/min/mg of protein (*p* = 0.0090) compared to the matching sham-operated control (0.43, [0.16–0.50]) in nmol O_2_/min/mg of protein, showing an increase in mitochondrial respiration not related to ATP production (Table 1). When we measured the maximal oxidative activity of the mitochondrial respiratory chain induced by FCCP (uncoupling respiration rate), we found that the median [interquartile range (IQR)] decreased in CLP groups throughout the course of sepsis but reached significance only at 48 h after the onset of CLP (−51%) (2.17, [1.89–3.39]) in nmol O_2_/min/mg of protein (*p* = 0.0034) compared to the matching sham-operated control (4.52, [4.23–5.18]) in nmol O_2_/min/mg of protein. The median [interquartile range (IQR)] of the specific cytochrome c oxidase (COX) activity was significantly lower throughout the course of sepsis at 8 h (−55%) (15, [13.20–20.83]) in nmol O_2_/min/mg of protein, (*p* = 0.0065), 24 h (−65%) (12.60, [1.89–3.39]) in nmol O_2_/min/mg of protein (*p* = 0.0019), and 48 h (−67%) (2.17, [1.89–3.39]) in nmol O_2_/min/mg of protein (*p* = 0.0035) compared to the matching sham-operated control at 8 h (33.86, [27.61–40.55]) in nmol O_2_/min/mg of protein, 24 h (36.90, [26.74–42.80]) in nmol O_2_/min/mg of protein, and 48 h (41.22, [25.44–45.46]) in nmol O_2_/min/mg of protein, respectively (Table 1). The RCR value (median ([interquartile range (IQR)]) was significantly lower in the CLP group at 48 h (1.85, [1.54–2.10]) (*p* = 0.0409) compared to the matching sham-operated control (17.06, [13.91–48.45]), showing the uncoupling of oxidative phosphorylation 48 h after the onset of sepsis. When comparing the RCR value (median [interquartile range (IQR)]) of CLP groups at 48 h (1.85, [1.54–2.10]) and 8 h (6.37, [3.11–28.19]), a statistically significant difference was noted (*p* = 0.0079). This confirms the preservation of mitochondrial efficiency in early sepsis. The higher value of RCR in the sham-operated control mice at 48 h after CLP shows their recovery from the surgical procedure.

Measures were performed on isolated liver mitochondria from the mice of sham-operated control and CLP groups 8, 24, and 48 h following surgery. Mitochondria were energized with glutamate (5 mmol L^−1^) and malate (2.5 mmol L^−1^) as respiratory substrates. Phosphorylating respiration (state 3 respiration rate) was initiated by the addition of 0.5 mmol L^−1^ of exogenous ADP and maximal oxygen consumption (an uncoupling respiration rate), which was initiated with 2 µmol L^−1^ FCCP in the presence of oligomycin. Non-phosphorylating respiration (state 4 respiration rate) was obtained by inhibiting ATP synthase with 3 µg mL^−1^ of oligomycin. Cytochrome c oxidase activity was assessed by adding 2 mmol L^−1^ of ascorbate plus 500 μmol L^−1^ N,N,N’N’-tetramethyl-phenylenediamine (TMPD) in the presence of 1.5 μmol L^−1^ of antimycin, oligomycin, and FCCP. RCR refers to the ratio of oxygen consumed after adding ADP to that which is consumed in the presence of oligomycin. Data are expressed as the median with 25th and 75th percentiles from five animals per group (n = 5) in each following period (8, 24, and 48 h after the onset of surgery). * *p* < 0.05 values are significantly different from the matching sham-operated control; ⸸ *p* < 0.05 values indicate a difference between 8 h and 48 h CLP groups. Statistical comparisons between groups were performed using the nonparametric Mann–Whitney test.

### 3.2. CLP Decreases Mitochondrial Calcium Retention Capacity and Induces Mptp Opening

**Figure 1:**
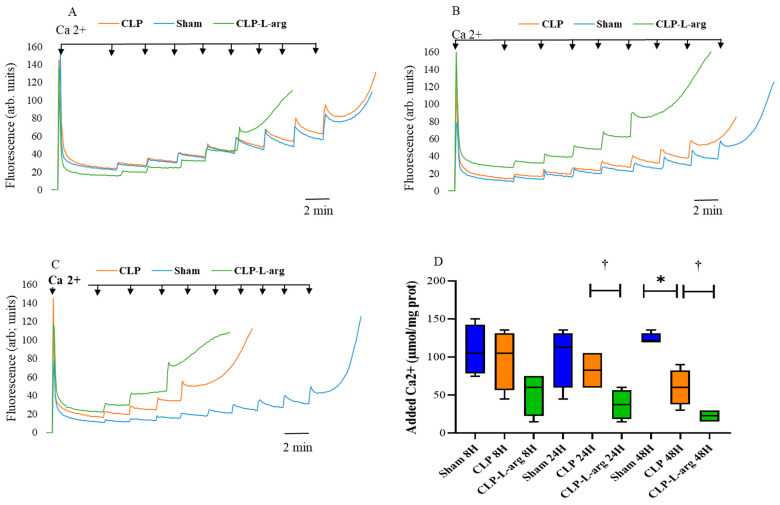
Response to Ca^2+^ challenge and L-arg treatment of mitochondria in CLP and sham-operated groups. Typical Calcium Green 5N tracing of liver mitochondria (1 mg mL^−1^) energized with glutamate (5 mmol L^−1^) and malate (2.5 mmol L^−1^) from matching sham-operated mice (blue line n = 4), CLP mice (orange line n = 4), and CLP mice treated with 1 mmol L^−1^ of L-arg (green line n = 4) at 8, 24, and 48 h after surgery (**A**–**C**). Arrows indicate addition of 15 µmol/mg of protein Ca^2+^ pulse. Tracings show progressive Ca^2+^ accumulation followed by release of accumulated Ca^2+^ secondary to PTP opening. (**D**) Average calcium retention capacity of mitochondria from matching sham-operated mice group (blue bar), CLP mice group (orange bar), and CLP mice group treated with 1 mmol L^−1^ of L-arg (green bar) at 8, 24, and 48 h after surgery. Data are expressed as median with 25th and 75th percentiles. Boxplots denote median and interquartile ranges, and whiskers denote min/max values from 4 animals per group (n = 4) in each following period (8, 24, and 48 h after the onset of surgery). * *p* = 0.0209 values indicate significant difference in CRC between CLP and matching sham-operated at 48 h; † *p* = 0.0433 values indicate a significant effect of L-arg on CRC of mitochondria in CLP mice groups at 24 h and 48 h (*p* = 0.0433). Statistical comparisons between groups were performed using nonparametric Mann–Whitney test.

As CLP can affect mPTP opening, we investigated the calcium retention capacity (CRC), another important mitochondrial function involved in sepsis [33]. The CRC (corresponding to the amount of Ca^2+^ required to induce mPTP opening) was measured by loading mitochondria with a train of Ca^2+^ pulses until a fast Ca^2+^ release occurred, which marked the onset of the mPTP. In these experiments, the addition of Ca^2+^ (15 µmol/mg protein) to isolated mitochondria from the liver resulted in a rapid increase in Calcium Green fluorescence, followed by a decline in the fluorescence intensity of the sensor (Figure 1A–C). This slower fluorescence, which decreased after the addition of Ca^2+^, was consistent with mitochondrial Ca^2+^ uptake [29,30]. After the sequential administration of pulses of Ca^2+^ (15 µmol/mg protein), the mitochondrial CRC was exceeded, and stored mitochondrial Ca^2+^ was released into the extramitochondrial space through the opening of the mPTP. As observed in the typical traces shown in Figure 1, mitochondria isolated from the CLP mice (orange color) and the corresponding sham mice (blue color) at 8 h (panel A) and 24 h (Panel B) resisted the addition of significantly higher amounts of Ca^2+^ pulses before the opening of the mPTP. There is no significant difference between the CLP and corresponding sham-operated group at 8 and 24 h post-surgery in the amount of calcium pulses added before the mPTP opens. This suggests that there is no difference in the CRC between CLP and the corresponding sham-operated group at 8 and 24 h after surgery. However, 48 h after CLP, the mouse liver mitochondria required significantly less calcium to be added to open the mPTP compared to the mitochondria of the corresponding sham-operated control mice. The median [interquartile range (IQR)] of the 48 h CLP group was 60 [37.5–82.5] µmol of calcium/mg of protein versus 120 [120–131] µmol of calcium/mg of protein (*p* = 0.0209). (Figure 1C,D). When comparing the 48 and 8 h CLP groups, the amount of calcium required to open the mPTP was smaller at 48 h (−44%), requiring 60 [37.5–82.5] µmol/mg of protein versus 105 [56.25–131.3] µmol/mg of protein (Figure 1C,D). Although there was no statistical difference between the two groups, the results show a tendency toward time-dependent increases in the susceptibility to mPTP opening from 8 h to 48 h post-CLP. (Figure 1A,C,D). The incubation of mitochondria with cyclosporin A can prevent mPTP opening and enhance CRC by about 30% in the control and CLP groups as usually described [29].

### 3.3. mtNOS Activation by L Arg Does Not Improve Mitochondrial Respiration or Prevent mPTP Opening

**Figure 2:**
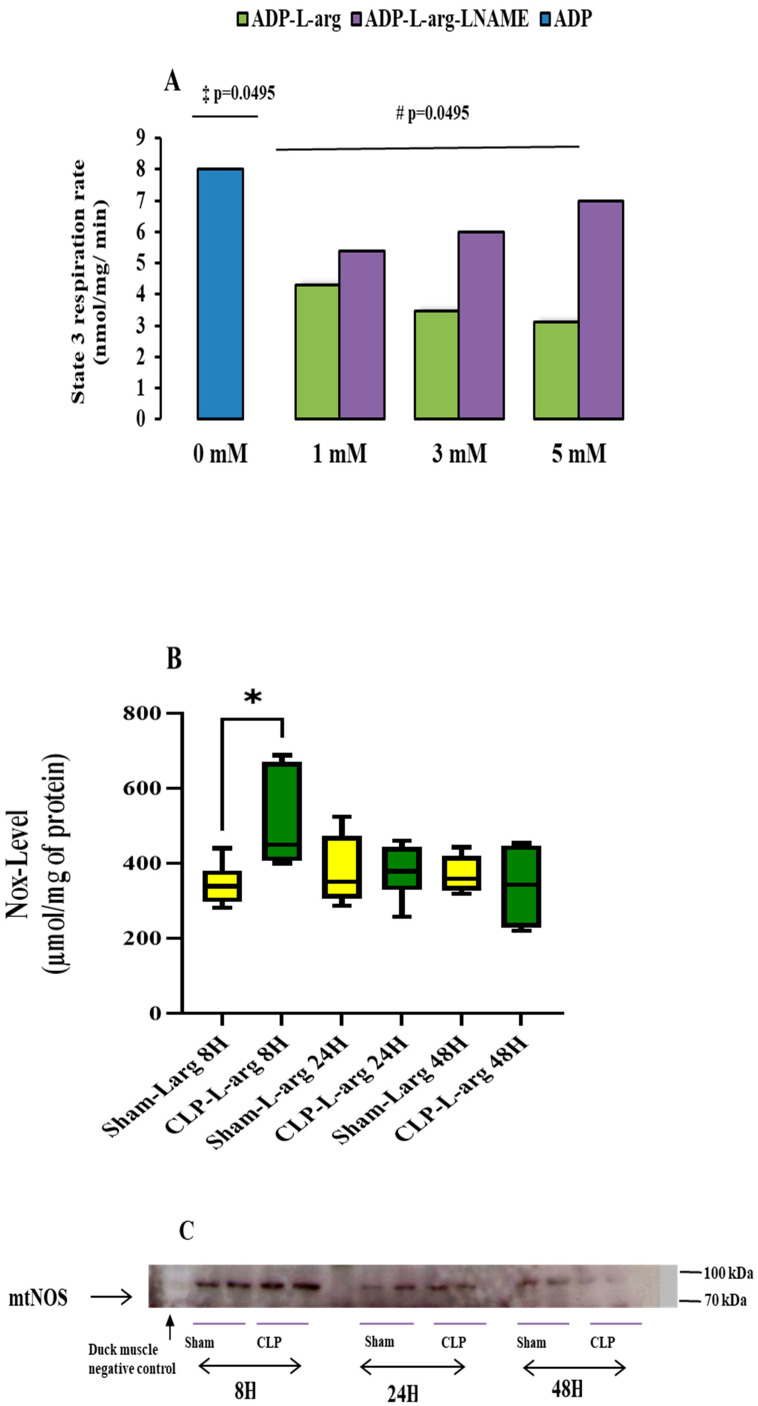
L-arg inhibited mitochondrial respiration, NO metabolites, and mtNOS content. (**A**). Respiration rates of liver mitochondria isolated from control mice in the presence of L-arg alone and L-arg plus L-NAME. Experiments were carried out in metabolic state 3 (0.5 mmol L^−1^ ADP) with energized mitochondria respiring on glutamate (5 mmol L^−1^) and malate (2.5 mmol L^−1^). Median (IQR) values for each group (n = 3 per group) are given in nmol O_2_/min/mg of protein: (ADP group) 8 [IQR 7–8], (ADP+ 1mM of L-arg) 4.3 [4–4.6], (ADP + 3 mM of L-arg) 3.45 [3.10–3.9], (ADP + 5 mM of L-arg) 3.10 [3–3.2], (ADP + 1 mM of L-arg + 1 mM L-NAME) 5.4 [5–5.8], (ADP + 3 mM of L-arg + 3 mM L-NAME) 6 [5.9–6.1], and (ADP + 5 mM of L-arg + 5 mM L-NAME) 7 [6–8.06]. Data are expressed as median (with 25th and 75th percentiles as noted above) from 3 independent measures in each group (n = 3). ‡ *p* = 0.0495 values are significantly different from ADP-L-arg groups; # *p* = 0.0495 values are significantly different from ADP-L-arg-L-NAME groups. Statistical comparisons between groups were performed using nonparametric Mann–Whitney test. (**B**) NOx-level determination by Griess reaction (see Section 2 Materials and Methods for details) in respiratory buffer containing mitochondria of CLP mice incubated with L-arg (orange box) and corresponding sham-operated mice (yellow box) after measurement of respiratory parameters, as described in Materials and Methods section. Data are expressed as median with 25th and 75th percentiles. Boxplots denote median and interquartile ranges, and whiskers denote min/max values from 5 animals per group (n = 5) in each following period (8, 24, and 48 h after onset of surgery). * *p* < 0.0037 values indicate higher levels of Nox- in CLP group compared to matching sham-operated group. Statistical comparisons between groups were performed using nonparametric Mann–Whitney test. (**C**) Western blot detection of mtNOS. Liver mitochondrial nitric oxide synthase was found to react with anti-NOS 2 [13]; we used primary antibodies of anti-mouse polyclonal anti-iNOS to identify mtNOS using Western blot standard techniques. Cold-acclimated Duck-muscle mitochondria were used as negative controls.

Leveraging our study on the activation of mtNOS in duck muscle with a Ki max calculated at 2.6 mmol L^−1^ and 2.8 mmol L^−1^ [14], we incubated the mitochondria of the control mice with an increasing dose of L-arg to observe the kinetic inhibitory action of mtNOS substrates upon state 3 respiration and determined the half-maximal inhibitory effects in control mice. The median reduction in the state 3 respiration rate (in nmol O_2_/min/mg of protein) in control mice(ADP-L-arg group) supplement with 1 mM of L-arg was (46%) 4.3 [4–4.6] (*p* = 0.0495), reaching 56% after 3 mM of L-arg 3.45 [3.10–3.9] (*p* = 0.0495) and 67% after 5 mM of L-arg 3.10 [3–3.2] (*p* = 0.0495) versus control mice non supplemented with (ADP group) 8 [IQR 7–8] (Figure 2A). The inhibitory effect of L-arg was fully reversed by incubating mitochondria with increasing doses of L-NAME (ADP-L-arg-L-NAME group). The median increase in the state 3 respiration rate (in nmol O_2_/min/mg of protein) in control mice with 1 mM of L-NAME was (+20%) 5.4 [5–5.8] versus 1 mM of L-arg alone (ADP-L-arg group) 4.30 [4–4.6] (*p* = 0.0495), +42% with 3 mM of L-NAME 6 [5.9–6.1] versus 3 mM of L-arg alone (ADP-L-arg group) 3.45 [3.10–3.9] (*p* = 0.0495), and +55% with 5 mM of L-NAME 7 [6–8.06] versus 5 mM of L-arg alone (ADP-L-arg group) at 3.10 [3–3.2] (*p* = 0.0495) (Figure 2A). The additional effect of L-arg and L-NAME determines the mtNOS functional activity on mitochondrial oxygen uptake [24], ensuring that the transport of L-arg into the mitochondria is unaffected by the mitochondrial isolation procedure. Therefore, we chose to use 1 mmol L^−1^ L-arg to study mitochondrial respiration and CRC. We acknowledge that a two-fold lower dose has been used in other studies [24,25], but higher doses have also been used in other animal species [14]. The incubation of mitochondria with L-arg did not improve the mitochondrial respiratory parameters in the CLP group compared to the matching sham-operated control (Table 2). In other words, L-arg did not reverse the drop in mitochondria respiration induced by CLP in mice.

**Figure 3:**
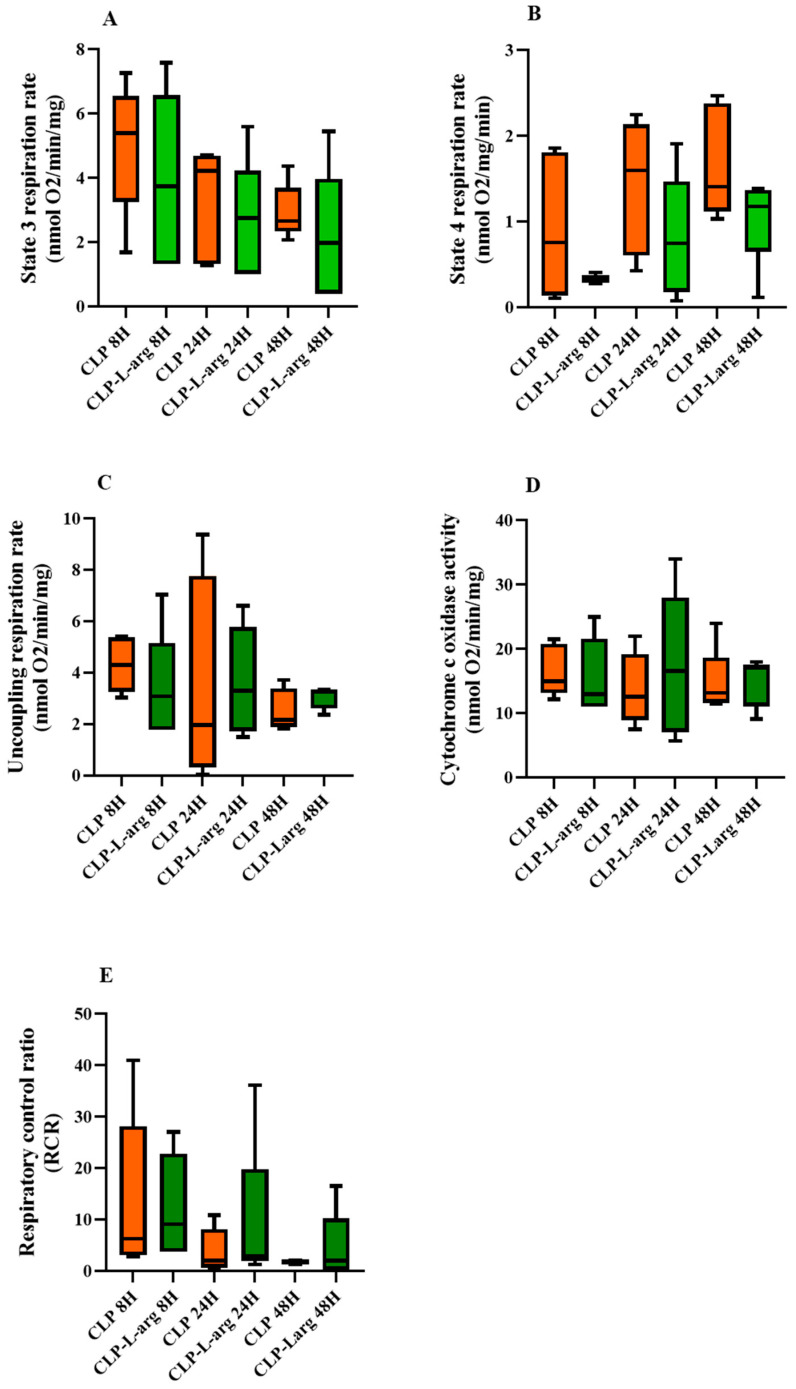
Effect of L-arg on liver mitochondrial oxygen uptake of septic mice. Liver mitochondria isolated from CLP mice at 8, 24, and 48 h following CLP were incubated with 1 mmol L of L-arg (green bar) or without L-arg (orange bar) at room temperature. Mitochondria were energized with glutamate (5 mmol L^−1^) and malate (2.5 mmol L^−1^) as respiratory substrates. Phosphorylating respiration (state 3 respiration rate), denoted by (**A**), was initiated by addition of 0.5 mmol L^−1^ of exogenous ADP, and maximal oxygen consumption (uncoupling respiration rate) denoted by (**C**) was initiated with 2 µmol L^−1^ FCCP in presence of oligomycin. Non-phosphorylating respiration (state 4 respiration rate) denoted by (**B**) was obtained by inhibiting ATP synthase with 3 µg mL^−1^ of oligomycin. Cytochrome c oxidase activity denoted by (**D**) was assessed by addition of 2 mmol L^−1^ ascorbate plus 500 μmol L^−1^ N,N,N’N’-tetramethyl-phenylenediamine (TMPD) in presence of 1.5 μmol L^−1^ antimycin, oligomycin, and FCCP. RCR refers to ratio of oxygen consumed after adding ADP to that consumed in presence of oligomycin denoted by the (**E**). Data are expressed as median with 25th and 75th percentiles. Boxplots denote median and interquartile range, and whiskers denote min/max values from 5 animals per group (n = 5) in each following period (8, 24, and 48 h after the onset of surgery). Statistical comparisons between groups were performed using nonparametric Mann–Whitney test.

When we compared the mitochondrial respiration of CLP mice treated with L-arg with those untreated at the same end time point, we found that L-arg showed decreased mitochondrial respiratory parameters in CLP-treated mice compared to untreated. However, the difference did not reach statistical significance (Figure 3). Additionally, 48 h after CLP, the decrease in state 4 respiration was more significant than that in state 3 respiration (−39% versus–12%), but this greater decrease in state 4 respiration did not significantly increase the RCR (mitochondrial efficiency) of CLP-treated mice 2.11 [0.32–10.31] compared to the untreated mice 1.85 [1.54–2.10]). In other words, L-arg did not improve mitochondrial efficiency 48 h after CLP, contrary to what was expected (Table 2). Although it was not the focus of our study, we noted that L-arg decreased cytochrome c activity in the sham-operated mice (Table 1 and Table 2).

Measures were performed on liver mitochondria from the mice of sham-operated and CLP groups at 8, 24, and 48 h following surgery. Mitochondria were energized with glutamate (5 mmol L^−1^) and malate (2.5 mmol L^−1^) as respiratory substrates in the presence of L-arg 1 mmol L^−1^. Phosphorylating respiration (state 3 respiration rate) was initiated by the addition of 0.5 mmol L^−1^ of exogenous ADP, and maximal oxygen consumption (uncoupling respiration rate) was initiated with 2 µmol L^−1^ of FCCP in the presence of oligomycin. Non-phosphorylating respiration (state 4 respiration rate) was obtained by inhibiting the ATP synthase with 3 µg mL^−1^ of oligomycin. Cytochrome c oxidase activity was assessed via the addition of 2 mmol L^−1^ of ascorbate plus 500 μmol L^−1^ of N,N,N’N’-tetramethyl-phenylenediamine (TMPD) in the presence of 1.5 μmol L^−1^ antimycin, oligomycin, and FCCP. RCR refers to the ratio of oxygen consumed after adding ADP to the amount consumed in the presence of oligomycin. Data are expressed as the median with 25th and 75th percentiles from five animals per group (n = 5) in each following period (8, 24, and 48 h after the onset of surgery). Statistical comparisons between groups were performed using the nonparametric Mann–Whitney test

When studying the effect of L-arg on the mitochondrial CRC of septic mice, we noted that the incubation of mitochondria in CLP groups with 1 mmol L^−1^ of L-arg decreased the amount of added calcium on mitochondria to open mPTP of CLP-L-arg groups by 50% at 24 h and 44% at 48 h, respectively. The median ([interquartile range (IQR)] of 24 h CLP-L-arg group was 37.5 [18.75–56.25]) in µmol of calcium/ mg of protein versus the 24 h CLP group with 82.5 [60–105] µmol of calcium/ mg of protein (*p* = 0.0433) (Figure 1D). The median ([interquartile range (IQR)] of the 48 h CLP-L-arg group was 22.5 [15–30] in µmol/mg of protein versus the 48 h CLP group with a median of 60 [37.5–82.5], *p* = 0.0433) (Figure 1D).

When comparing 48 h of CLP-L-arg with 8 h CLP-L-arg, we found that the amount of calcium added to promote pore opening was lower in the 48 h group by 42%, (22.5 [15–30] µmol/ mg of protein versus 60 [22.5–75]) in µmol/mg of protein; however, the difference did not reach statistical significance (Figure 1D).

### 3.4. Effect of Sepsis on Mitochondrial mtNOS Content and Nitric Oxide Metabolites Level

The NOx- levels were higher in respiratory buffers containing the mitochondria of CLP mice incubated with L-arg. The median Nox- levels of the CLP-L-arg group at 8 h was 450 [375–670] in µmol/mg of protein (+47%) versus 381 [296–381] in matching the Sham-L-arg group (*p* = 0.037). This then decreased overtime by 25% and 33% at 24 and 48 h compared to the matching sham-operated group (Figure 2B).

Figure 2C provides evidence of mtNOS on the mitochondria pellet. The absence of reactivity with the mitochondrial pellet of the acclimated duck shows the specificity of the reaction. Given that we obtained the functional activity of the enzyme, we did not consider it useful to quantify the amount of protein.

## 4. Discussion

Here, we describe the effect of L-arg, an mtNOS activator, on liver mitochondrial respiration and permeability transition pore opening during sepsis. We determined the time in which intra-mitochondrial NO synthesis would be helpful for mitochondrial respiration and CRC during the course of sepsis. We found that L-arg-induced mtNOS activation inhibited mitochondrial respiration and decreased CRC throughout the time course of sepsis. This effect worsened at 48 h of sepsis, where L-arg showed no benefit for mitochondrial function.

We used a standard CLP mouse model of sepsis, which caused 75% mortality within 2 days [26,27,28], with most deaths occurring before 48 h. Based on this observation and previous reports [19], we assumed that the mice surviving at 48 h after CLP without surgical resection of cecum or antibiotic infusion could be considered to have survived sepsis or exhibited chronic sepsis [34,35]. Mice surviving sepsis might then enhance their mitochondrial function [22,23], which was not the case in this study. In the present study, 48 h after CLP, the mitochondrial respiratory parameters were profoundly altered (Table 1). The phosphorylating respiratory rate (state 3 respiration), maximal oxidative rate (uncoupling state), and cytochrome-c oxidase (COX) activity were significantly reduced, reflecting the inhibition of ATP turnover and the substrate oxidation and dysfunction of ETC [36]. This drop in mitochondrial oxidative capacity was associated with an increase in the non-phosphorylating respiratory rate (state 4 respiration). Although the phosphorylating respiratory rate decreases early in sepsis [22], the occurrence of an increase in the non-phosphorylating respiratory rate 48 h post-CLP surgery results in a decrease in RCR. Such mitochondrial respiration abnormalities have previously been ascribed to an uncoupling of liver mitochondrial respiration and ADP phosphorylation in the late stages of sepsis in rats [19]. It is commonly stated that an increase in non-phosphorylating respiration corresponds to a leak of protons through the inner membrane [36]. However, in two consecutive studies [7,19], we did not find an increase in proton leaks in CLP rats, meaning that the proton conductance of the mitochondria inner membrane is not affected by CLP [22]. We, therefore, concluded that a slippage of COX and ATP synthase was responsible for the decrease in oxidative phosphorylation efficiency in sepsis [22]. Unlike other chronic conditions [20,21], a slip in COX was not accompanied by an improvement in oxidative phosphorylation efficiency. The present increase in state 4 respiration and decrease in the RCR could be explained by the slight protective uncoupling of oxidative phosphorylation to decrease detrimental ROS generation [37]. This happens in sepsis via the transient activation of uncoupling protein 2 [19]. Since uncoupling decreases proton motive force and ATP production (state 3 respiration), it facilitates electron flow from reduced dinucleotides to oxygen, therefore reducing the increased production of superoxide and decreasing cellular oxygen tension, which is toxic (via an increase in basal oxygen consumption) [37]. Moreover, this phenomenon also aligns with the opening of the mPTP [38]. This is supported by our data, which show a concomitant increase in mitochondrial state 4 oxygen consumption, a decrease in RCR, and an opening of the mPTP 48 h after CLP surgery. However, one of the consequences of mPTP opening is the dissipation of the proton motive force (the collapse of the mitochondrial membrane potential), which results in the persistent uncoupling of oxidative phosphorylation [38]. The opening of the mPTP is triggered in vitro by a calcium overload, but its sensitivity is dependent on various conditions [39], including oxidative and nitrosative stress, which are present from the early stages of sepsis [7,19]. In addition, any impairment of mitochondrial ETC leading to a decrease in ATP synthesis as cytochrome-c oxidase (COX) dysfunction (Table 1) could reduce mitochondrial calcium uptake and storage [40]. Given the important role of COX in ATP synthesis and its early dysfunction found in the present study, it seems logical that we found aberrant mitochondrial calcium homeostasis. However, the drop in the calcium retention capacity only occurred 48 h after CLP surgery (Figure 1). This decrease in calcium storage by mitochondria is concomitant with a decrease in the RCR. This observation describes a succession of events where early COX dysfunction precedes the mPTP opening and, finally, persistent mitochondrial uncoupling.

We then considered whether, by applying a constraint on the cytochrome-c oxidase of mitochondria from CLP mice, we could improve mitochondrial ATP generation and delay mPTP opening as described in previous studies on healthy rat mitochondria [16,17]. We chose L-arg as the NO donor, which can be used without harm under conditions of a significant reduction in nitric oxide [41]. L-arg is known to be a substrate of mitochondria nitric oxide synthase (mtNOS) and is converted to NO and L citrulline [13]. This conversion makes L-arg an endogenous source of NO, including in mitochondria [13]. Based on our previous work [14], we chose to use 1 mM of L-arg to study mitochondrial respiration and the mPTP. As shown in Figure 2A, 1 mM of L Arg inhibited mitochondrial respiration by 46% in a non-saturant fashion in the control mice. Applying an NOS inhibitor (i.e., L-NAME) mitigated the effect of L-arg on mitochondrial respiration, indicating that 1 mM of L-arg, as used in this study, was probably the physiological level of mtNOS activity [42]. An L-arg infusion on the mitochondria of the CLP mice groups was followed by an increase in mitochondrial NO production (NOx- levels) at 8 h in the CLP group, corresponding to a peak in NOS activity and pattern of NO production in CLP sepsis models [43]. Contrary to Fontaine [17] and Dinnyk [16], the application of L-arg on mitochondria from CLP animals in this study did not improve mitochondrial efficiency (Table 2) or delay mPTP (Figure 1). This likely happened due to the early and consistent inhibition of cytochrome-c oxidase (Table 1) and its depletion [7,19], resulting from CLP. Nevertheless, the negative effect of NO on mitochondrial function and the mPTP found in the present study was significant only at the 48th hour post-CLP, when COX activity (Table 1) and mitochondrial NO synthesis (Nox- level) (Figure 2B) were already reduced. This observation highlights that a mediator other than NO itself affects mitochondrial efficiency and the mPTP 48 h after CLP. We suggest here that several hours of exposure of mitochondrial components to endogenous NO and derivates [6,10,43] in vivo can damage mitochondrial proteins [44,45], decreasing ATP synthesis [46]. Considering that the over-expression of iNOS in other experimental models delayed the mPTP [47], the present study shows that the activation of nitric oxide production 48 h after the onset of CLP increases the sensitivity of the mPTP. This contradictory effect highlights other aspects of NO and derivates on mitochondria, including ATP synthesis in particular and the mPTP several hours after the onset of infection [45,46].

We also provide physical evidence of the presence of mtNOS on mitochondria pellets. Indeed, we incubated our mitochondria with anti-iNOS antibodies [13]. We showed the presence of mtNOS in both the CLP and sham groups, regardless of the period following surgery. We also added mitochondria from duck muscles [14] as a negative control for which the same antibody showed no reactivity.

## 5. Limitations of Our Study

A limitation of our study is the ex vivo measurement of oxygen consumption at the non-physiological level of oxygen. However, Clark-type electrodes have been used for over 50 years, leading to an intimate understanding of mitochondrial respiratory function. The absence of ROS and RNS quantification and the use of 1 mM of L-arg were also limitations of this study. We did not measure ATP production; however, in our previous study, the decrease in ATP production was associated with a decrease in state 3 respiration. Further studies are needed with a lower amount of L-arg on mitochondria with the same experimental model.

## 6. Conclusions

In this study, we confirmed an uncoupling of oxidative phosphorylation 48 h after CLP that could be explained by COX dysfunction and the opening of mPTP. However, L-arg did not restore mitochondrial coupling or prevent the onset of the mPTP. L-arg would perhaps not be a suitable treatment to cure mitochondrial dysfunction in sepsis.

## Figures and Tables

**Table 1:** Mitochondrial respiratory parameters. Phosphorylating respiration (State 3 respiration rate); non-phosphorylating respiration (State 4 respiration rate); FCCP-induced maximal oxygen consumption (uncoupling respiration rate); cytochrome c oxidase activity; and respiratory control ratio (RCR). * *p* < 0.05 values are significantly different from the matching sham-operated control; ⸸ *p* < 0.05 values indicate a difference between 8 h and 48 h CLP groups.

	8 h		24 h		48 h	
	CLP	Sham	CLP	Sham	CLP	Sham
State 3 Respiration Rate nmol O_2_/min/mg	5.40 [3.26–6.55]	6.53 [3.49–9.84]	4.23 [1.33–4.70] *	6.66 [5.13–10.02]	2.66 [2.43–3.70] *⸸	6.24 [6.08–9.62]
State 4 Respiration Rate nmol O_2_/min/mg	0.76 [0.14–1.81]	1.07 [0.31–1.49]	1.60 [0.60–2.13]	0.53 [0.34–1.59]	1.41 [1.12–2.38] *	0.43 [0.16–0.50]
Uncoupling Respiration Rate nmol O_2_/min/mg	4.30 [3.25–5.37]	4.67 [3.76–6.82]	1.97 [0.32–7.78]	4.89 [3.84–7.82]	2.17 [1.89–3.39] *	4.52 [4.23–5.18]
Cytochrome C oxidase activity nmol O_2_/min/mg	15 [13.20–20.83] *	33.86 [27.61–40.55]	12.60 [8.95–19.21] *	36.90 [26.74–42.80]	13.21 [11.63–18.73] *	41.22 [25.44–45.46]
Respiratory Control Ratio	6.37 [3.11–28.19]	6.54 [5.81–13.29]	2.09 [0.74–8.18]	9.38 [6.16–19.59]	1.85 [1.54–2.10] *⸸	17.06 [13.91–48.45]

**Table 2:** Effect of L-arg on mitochondrial respiratory parameters. Phosphorylating respiration (State 3 respiration rate), non; phosphorylating respiration (State 4 respiration rate), FCCP; induced maximal oxygen consumption (uncoupling respiration rate), cytochrome c oxidase activity, and respiratory control ratio (RCR).

	8 h		24 h		48 h	
	CLP	Sham	CLP	Sham	CLP	Sham
State 3 Respiration Rate nmol O_2_/min/mg	3.75 [1.32–6.58]	4.27 [0.945–8.29]	2.76 [1.01–4.23]	4.74 [3.30–5.78]	1.99 [0.41–3.98]	4.59 [2.20–7.23]
State 4 Respiration Rate nmol O_2_/min/mg	1.41 [1.12–2.38]	1.20 [0.44–2.20]	0.75 [0.18–1.47]	0.15 [0.06–0.061]	1.18 [0.65–1.37]	0.68 [0.46–1.08]
Uncoupling Respiration Rate nmol O_2_/min/mg	3.09 [1.77–5.17]	3.93 [2.02–8.50]	3.30 [1.72–5.79]	4.23 [2.21–4.72]	3.29 [2.61–3.32]	4.37 [2.33–5.31]
Cytochrome C oxidase activity nmol O_2_/min/mg	13 [11–21.64]	14.88 [12.72–27.85]	16.6 [7.10–28]	18.29 [17.32–23.45]	17.1 [11.13–17.55]	18.50 [14.97–26]
Respiratory Control Ratio	9.41 [3.77–22.84]	2.26 [1.30–12.51]	2.93 [2.01–19.85]	22 [14.19–74.06]	2.11 [0.32–10.31]	5.59 [4.32–9.32]

## Data Availability

The data presented in this study are available on request from the corresponding author.

## Abbreviations

ATP: adenosine triphosphate CLP: Cecal ligation and puncture. COX: cytochrome-c oxidase. ETC: electron transport chain. FCCP: carbonyl cyanide-p-tri-fluoro-methoxy-phenyl-hydrazone. L-arg: L arginine. L-NAME: NG-nitro-L-arginine methyl ester. NO: nitric oxide. mtNOS: mitochondrial nitric oxide synthase. mPTP: mitochondrial permeability transition pore. ROS: reactive oxygen species, TMPD: N,N,N’,N’-tetramethyl-1,4-benzenediamine dichloride.
